# Supplementary material for: Injuries, Pain, and Catastrophizing Level in Gymnasts: A Retrospective Analysis of a Cohort of Spanish Athletes
Source: Healthcare (Basel). 2022 May 12;10(5):890. doi: 10.3390/healthcare10050890 (PMC9141380; doi:10.3390/healthcare10050890)
Supplement: Supplementary file 1 [file healthcare-10-00890-s001.zip › healthcare-1701726-supplementary.pdf]

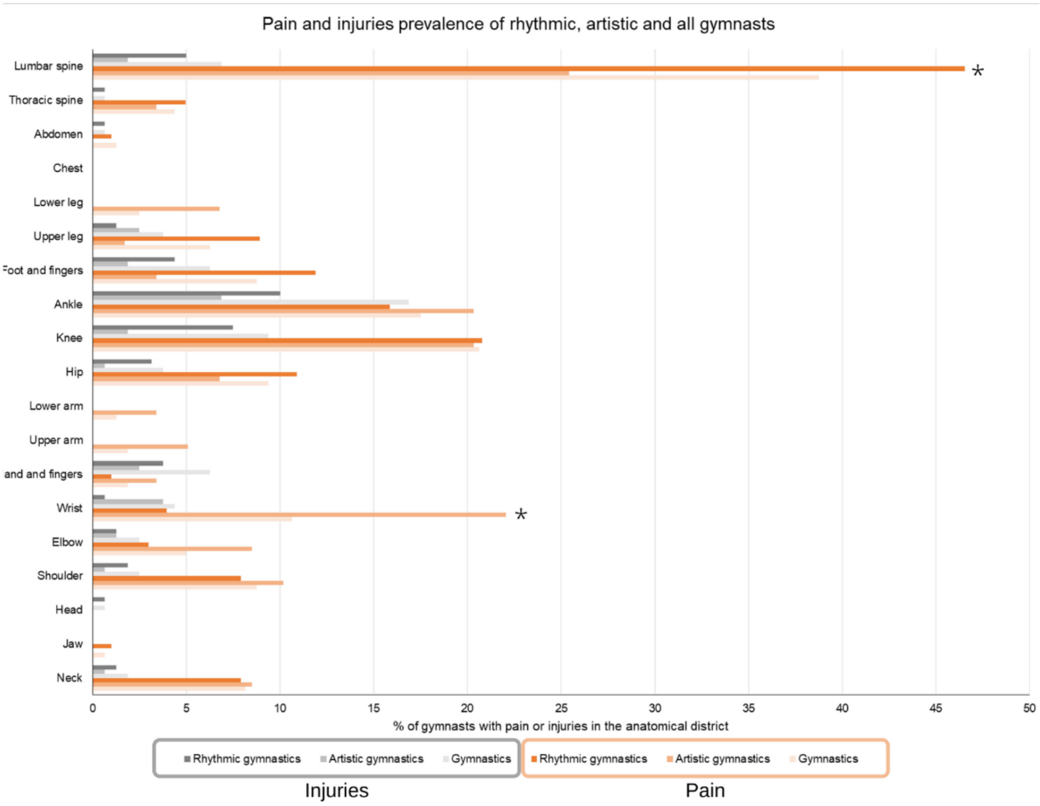

Figure S1. Prevalence of pain and injuries of rhythmic, artistic, and all gymnasts.

Table S1. Socio-demographic characteristics, injuries, pain level and catastrophizing level for FEMALE gymnasts.

	**Total (n =150)**	**Artistic (n = 50)**	**Rhythmic (n = 100)**
Age (yrs)[a]	16.7 (2.8)	16.2 (3.5)	16.9 (2.6)
BMI (kg/m^2)[a]	20.2 (2.3)	20.6 (2.3)	20.0 (2.2)
Experience (yrs)[a]	9.5 (4.0)	8.6 (4.6)	9.8 (3.5)
Competition Level (% elite)	18.0	34.0	10.0
Training volume (hrs/wk)	17.1 (12.4)	20.0 (16.2)*	15.6 (9.8)
Injured in this season (%)	48.0	44.0	50.0
Injuries (n)	104	37	67
PCS (total score)[a]	21.4 (10.2)	20.4 (9.8)	21.9 (10.5)
PCS (Rumination) (score)[a]	8.3 (4.1)	8.2 (4.0)	8.3 (4.1)
PCS (Helplessness) (score)[a]	8.2 (4.7)	7.4 (4.3)	8.6 (4.8))
PCS (Magnification) (score)[a]	4.9 (2.8)	4.8 (2.9)	5.0 (2.8)
Pain existence (%)	73.3	66.0	77.0

Pain in 1 district (%)	29.3	24.0	32.0
Pain in 2 district (%)	27.3	22.0	30.0
Pain in ≥3 district (%)	16.7	20.0	15.0
Pain Peak (0-10 score)[a]	4.3 (3.1)	3.8 (3.1)	4.6 (3.1)

PCS, pain catastrophizing scale; [a], values are given as mean (standard deviation); *, significant differences compared to rhythmic gymnastics ($p < 0.05$).
